# High particle variability across siliconized and oil-free syringes and needles from the same lots

**DOI:** 10.1038/s41598-021-84158-0

**Published:** 2021-02-25

**Authors:** Lydianne Lumack do Monte Agra, Natasha Ferreira Santos da Cruz, Vaida Linkuviene, John F. Carpenter, Michel Eid Farah, Gustavo Barreto Melo, Maurício Maia

**Affiliations:** 1 Hospital de Olhos de Sergipe, Rua Campo do Brito, 995, Aracaju, SE Brazil; 2 Federal University of Sergipe, Av Marechal Rondon, s/n, São Cristovão, SE Brazil; 3 Department of Ophthalmology, Federal University of São Paulo, Rua Botucatu, 806, São Paulo, SP 04026-062 Brazil; 4 Department of Pharmaceutical Sciences, University of Colorado, Anschutz Medical Campus, Aurora, CO USA

**Keywords:** Health care, Medical research

## Abstract

Previous studies have reported silicone oil (SO) applied to needles and syringes in the vitreous of patients after intravitreal injections. We evaluated four syringes (SR 1-mL insulin, Saldanha-Rodrigues; BD 1-mL Tuberculin Slip Tip, Becton–Dickinson; BD Ultra-Fine 0.3 mL, HSW Norm-Ject Tuberculin, Henke Sass Wolf) and 10 needles (BD PrecisionGlide 27- and 30-gauge (G); BD Eclipse and JBP Nanoneedle 27-, 30-, 33- and 34-G; TSK Invisible Needle and 27 and 30-G Steriject Control Hub). The protein-free buffer samples injected into the syringes and needles under study were collected in an Eppendorf tube and taken to Flow imaging microscopy, that characterized the concentration and morphology of the microsized particles. The number of particles was analyzed. The coefficients of variation (CV) were the primary outcome. The Feltz and Miller test compared the CVs. The significance level was 5%. Numerous particles and high CVs were associated with both devices, needles and syringes; the comparisons among them did not reach significance. The BD Ultrafine 0.3 mL syringe (149.7%) had the highest CV and the SO-free HSW Norm-Ject (66.4%) syringe the lowest, and the TSK Invisible needle (149.5%) had the highest and the BD Precision Glide 30G needle (35.9%) needle the lowest. In conclusion, particle release, including those with SO morphology, varied greatly among instruments, even from the same lots, which is relevant considering that fewer particles are injected into some eyes compared with others.

## Introduction

The pharmaceutical industry has used silicone oil for half a century and its lubricating properties vary greatly in the syringes and needles for medical use, including those used for intravitreal injections (IVIs), which are now the most common eye procedures worldwide^1^.

Device siliconization is an important step in the production of syringes and needles and one of the main goals is to achieve a uniform amount of SO in each unit^2^. The purpose of syringe siliconization is to create the most even anti-friction coating possible along the entire length of the syringe to minimize break loose and gliding forces when the plunger stopper is deployed. The coating should guarantee better syringe performance and consistent injection force^3^. Siliconized needles tend to slide better through the eye wall and cause less tissue damage as a result of the smaller scleral opening, which decreases drug reflux and ensures greater comfortable for the patient^4^.

Despite these advantages of SO, previous studies have shown that the SO applied to these devices are found as droplets in the vitreous of patients who undergo IVIs^5–7^. These droplets in the vitreous can be asymptomatic, but it is not uncommon for patients to have persistent floaters, which can eventually require vitrectomy, with its associated risks. In addition, SO has been speculated to be involved in some cases of noninfectious endophthalmitis after antiangiogenic IVIs^8^.

Considering this, the clinical importance of achieving the most efficient lubrication possible using minimal SO to achieve uniform coating of the materials is imperative. While the physicians administering IVIs expect that syringes and needles have a consistent and predictable amount of lubricant, high variability in that amount of SO can lead to heterogenous release of particles into the vitreous. The current study assessed both the levels of particles, including SO droplets, released by some of the most popular syringe and needle models used by ophthalmologists worldwide, and their variability across samples from the same lot.

## Results

Forty syringes, 10 of each model from a specific manufacturing lot, were assessed. The levels of particles (including those with SO morphology) released from each model are listed in Table 1. Large numbers of particles and a significantly high coefficients of variation (CVs) were seen. The CV was highest in the BD Ultrafine 0.3 mL (149.7%) and lowest in the SO-free HSW Norm-Ject (66.4%) syringes. The BD Tuberculin and SR syringes had CVs of 111.6% and 118%, respectively.

**Table 1:** Summary of particle numbers/mL by syringe model.

Model	Mean	Standard deviation	Minimum	Maximum	P25	Median	P75	N
BD Tuberculin	6393.3	7139.3	1733.3	24,173.3	2368.3	3120.0	8230.0	10
BD Ultra-Fine	2,319,655.3	3,473,855.8	16,813.3	10,340,786.6	48,223.3	671,363.3	4,134,740.0	10
HSW Norm-Ject	16,057.3	10,665.9	3773.3	34,166.6	8310.0	12,620.0	25,221.6	10
SR	16,869.3	19,919.3	3373.3	60,593.3	5955.0	8336.6	20,121.6	10

**Figure 1:**
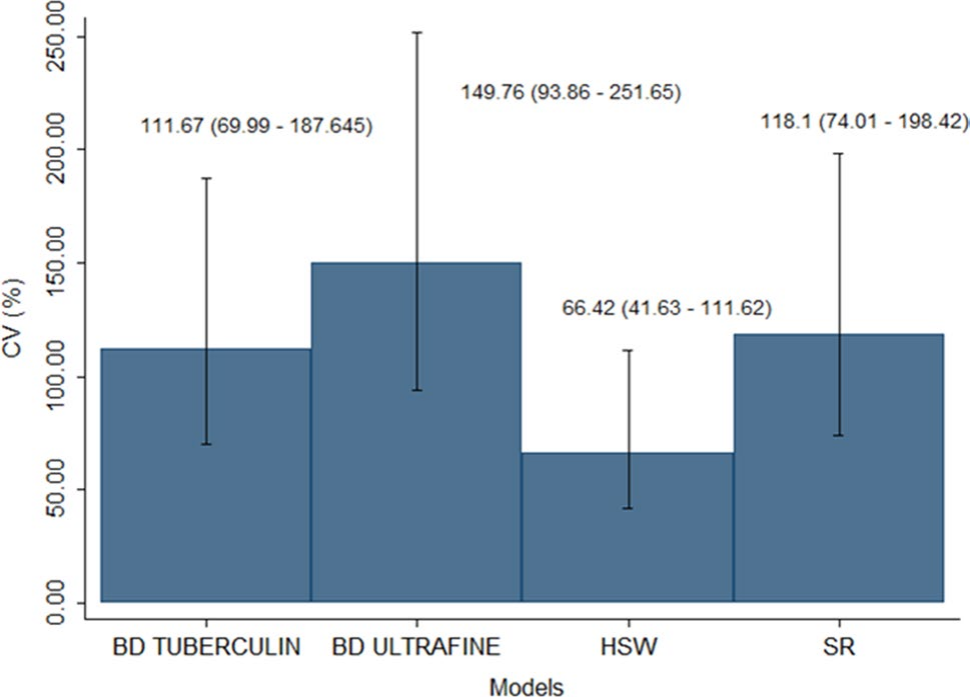
Coefficients of variation (CV) of the syringe models. The Feltz and Miller asymptomatic test was used for CV comparisons—Chi2(3) = 1.47 (*p* = 0.689).

With regard to particle counting the syringes results varied as follows: BD 1-mL Tuberculin Slip Tip (from 1733.3 to 24,173.3 particles/mL), BD Ultra-Fine 0.3 mL (from 16,813.3 to 10,340,786.6), HSW Norm-Ject Tuberculin (from 3773.3 to 34,166.6) and SR 1-mL insulin (from 3373.3 to 60,593.3). This information can be found in Table 1. These differences among the models were not significant (Fig. 1).

**Figure 2:**
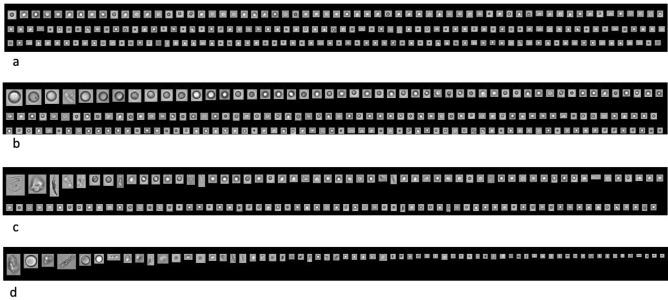
Illustrative examples of particles from the syringes seen in flow imaging microscopy images. **a** Circular particles from the HSW Norm-Ject, a silicone oil (SO)-free syringe, suggest oil-like lubricating components. **b** The BD Ultra-Fine, a siliconized syringe, with circular particles consistent with SO droplets. **c** Particles from the BD-Tuberculin syringe suggest fibrils. **d** Particles from the Saldanha Rodrigues, a siliconized syringe.

The particles found in samples can include fibers, plastic, and oil-like lubricating components, which have circular images (Fig. 2c). A wide variety of such particles were found in the all syringes studied, both siliconized and oil-free models. From these siliconized syringes, the vast majority of particles had circular morphologies in flow microscopy images, consistent with SO droplets (Fig. 2a,b,c,d).

A total of 97 needles units were assessed, 10 samples of each model, except for the TSK Invisible Needles, for which we assessed seven samples.

The particle number/mL in the needle samples, in turn, varied as follows: BD Eclipse (from 0 to 9200), BD PrecisionGlide 27G (from 1800 to 7620), BD PrecisionGlide 30G (from 1450 to 6600), JBP Nanoneedle 27G (from 1950 to 6830), JBP Nanoneedle 30G (from 1330 to 6700), JBP Nanoneedle 33G (from 1380 to 9850), JBP Nanoneedle 34G (from 1310 to 9270), TSK Invisible Needle (from 0 to 7870), TSK 27G (from 0 to 5130) and TSK 30G (from 0 to 6530). The data are presented in Table 2.

**Table 2:** Summary of particle numbers/mL by needle model.

Model	Mean	Standard deviation	Minimum	Maximum	P25	Median	P75	N
BD Eclipse	4641	3439	0	9200	1350	4380	8530	10
**BD PrecisionGlide**								
27-G	4006	1671	1800	7620	2760	3930	4990	10
**BD PrecisionGlide**								
30-G	4596	1653	1450	6600	3810	4835	6050	10
JBP 27-G	4352	1580	1950	6830	3550	4330	5470	10
JBP 30-G	2864	2058	1330	6700	1500	1765	4200	10
JBP 33-G	6294	2968	1380	9850	4720	6900	8930	10
JBP 34-G	4813	3326	1310	9270	1450	3935	8670	10
TSK Invisible	1886	2820	0	7870	0	1330	2670	7
TSK Steriject 27-G	1942	1788	0	5130	0	1460	3110	10
TSK Steriject 30-G	2475	2165	0	6530	1330	1915	3930	10

**Figure 3:**
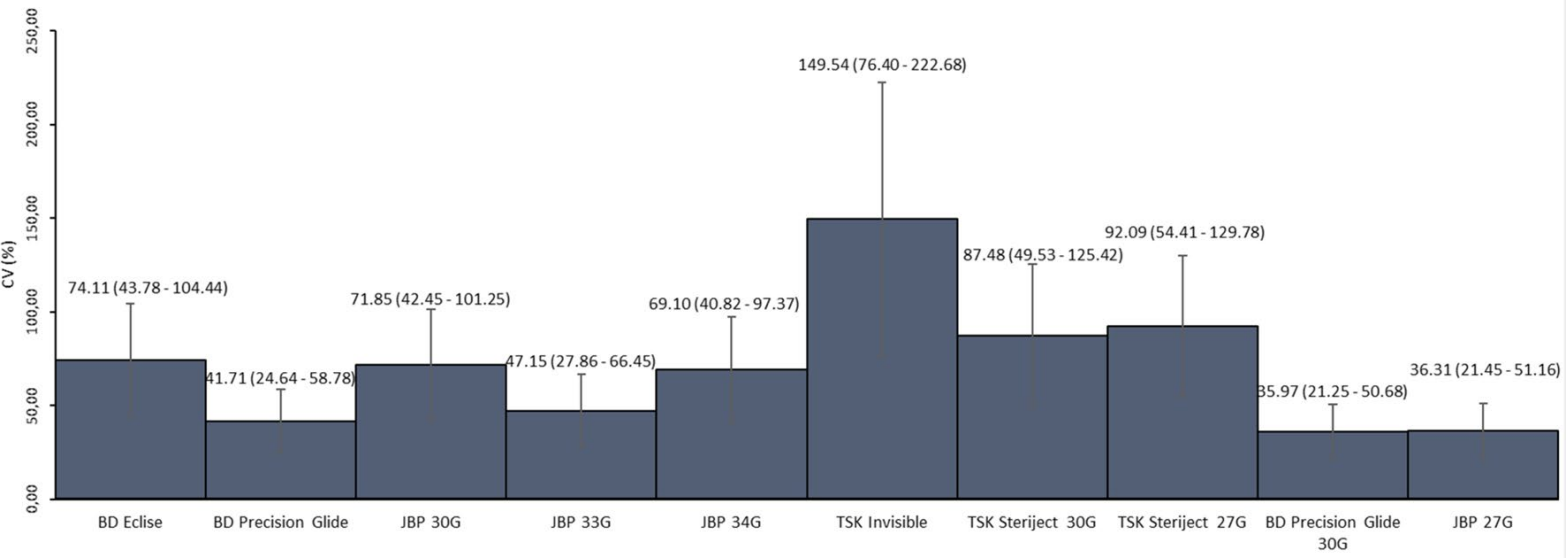
Coefficients of variation (CV) (%) of the needle models.

As with the syringes, the CVs were very high. The TSK Invisible Needle had the highest CV (149.5%), while the BD PrecisionGlide 30G had the lowest CV (35.9%). Nevertheless, no significant difference was seen in the variability of the 10 models evaluated (Fig. 3).

## Discussion

The exponential increase in the number of IVIs performed worldwide during the last decade has focused attention on the materials used during these procedures, especially the syringes and needles. Among the various stages in the material manufacturing process, the siliconization of their surfaces is crucial, since a lubricated device reduces the force required for its use and makes the procedure more comfortable and safer for the doctor and patient. Despite siliconization, however, SO has been found in the vitreous of several patients, which can have clinical implications, such as floaters and intraocular inflammation^9^.

We reported previously that the needles and syringes tended to be coated with SO, which may ultimately be injected into patients’ eyes^9–11^. By studying different syringe and needles samples from the same brand and lot in the current study, we identified great variability in the numbers of particles released. The CVs, ranging from 66 to 150% for syringes and from 36 to 150% for needles, indicated that the marked variations do not depend on the model or whether it is SO-free.

The use of CVs to measure the accuracy and precision of equipment and analytical methods, which is common in academic studies, is a reliable predictor that is easily interpreted in clinical practice. Regarding particle release by syringes and needles from the same lot, few data have been published. Yet, it is well known that a lower CV implies better equipment precision. Values lower than 10% generally are considered desirable. Therefore, CVs ranging from 36 to 150% across the different models tested in this study, for example, imply that the uniformity of these devices is highly questionable^12–14^.

The BD Ultra-Fine syringe had the highest variability and the highest mean particle count and is the only model among those tested that is equipped with a staked-in needle. The HSW Norm-Ject had the lowest variation among its samples; this is a SO-free syringe and the particles found in this sample can include fibers, plastic, and oil-like lubricating components, as described before. which have circular images (Fig. 3C) A wide variety of such particles were found in the other syringes studied. The BD Tuberculin and SR syringes, both siliconized, showed a very similar types of particle morphology. From these siliconized syringes, the vast majority of particles had circular morphologies in flow microscopy images, consistent with SO droplets (Fig. 3A,B,C,D).

Similar to the syringe findings, the variability in the particles released in the needles from the same lot is of clinical relevance, regardless of the model analyzed. The BD PrecisionGlide 30G needle (CV, 35%) had the least variability and yet the CV was high, in that such variability greatly hinders reliable prediction of particle release under normal clinical conditions. Along those same lines, CVs as high as 71.8% (JBP 30-G), 87.4% (TSK Steriject), and 149.5% (TSK Invisible Needle) cause uncertainty about the outcomes of a procedure such as an IVI.

At this point, a consideration about the release of silicone oil by the needles should be made. The source of the silicone oil in the eye from the neddle is more likely due to friction during globe penetration, since the siliconization of these devices is made on the outer wall, to avoid pain and make a soft penetration. Nevertheless, part of this silicone oil can be found on the inner wall of the needles (the part of the needle from which our samples were obtained) and that's the part directly injected into the vitreous, unlike the oil of the outer wall, which is probably mostly retained externally to the eye. However, studies that evaluate the outer wall of the needles and the release of particles due to friction during globe penetration might help to better clarify the real role of the needles in the release of particles during the IVI.

The morphologic considerations regarding particles released from needles are similar to particles released from syringes: circular images are consistently seen with SO. Other particles such as fibers and plastic also were seen in the needle samples.

Thus, we observed that all studied syringes and needles exhibited great variability in the release of particles, including SO, across samples of the same lot, with no significant differences among the assessed models.

In conclusion, ophthalmologists who perform IVIs should be aware of this wide variability in particles from syringes and needles due to the potential clinical implication of particles, in particular SO, injected into the vitreous of their patients. Better control is needed in the siliconization process and particle loads from these devices, including the SO-free ones, which will provide greater safety and increase physicians confidence about their clinical outcomes.

## Methods

Four syringes were studied: the SR 1-mL insulin (Saldanha-Rodrigues, Pedro Juan Caballero**,** Paraguay, lot #F589T), BD 1-mL Tuberculin Slip Tip (Becton–Dickinson and Co., Frankli Lakes, NJ, USA, lot #9086864, ref #309659), BD Ultra-Fine 0.3-mL Short Needle with a half-unit-scale (Becton–Dickinson and Co., lot #7100846, ref #328440), and HSW Norm-Ject Tuberculin (Henke Sass Wolf, Tuttlingen, Germany, lot #18L15C8, ref #4010-200V0). Except for the BD Ultra-Fine, which has a staked-in needle, the syringes were tested without an attached needle.

Ten needles were assessed: BD PrecisionGlide 27-(G) (lot #9077702) and 30-(G) (lot #9093839) and BD Eclipse 30-G (lot #8362592) (Becton–Dickinson and Co., Franklin Lakes, NJ, USA), JBP Nanoneedle 27-G (lot #4964), 30-G (lot #4936), 33-G (lot #4967), 34-G (lot #D4910) (Feel Tech Co., Cheonan-si, South Korea), and TSK Invisible Needle (lot #204174) and TSK Steriject Control Hub 27-G (lot #204210) and 30-G (lot #204326) (TSK Laboratory, Tochigi, Japan).

The concentration, size distribution, and morphology of the microsized particles were characterized using flow imaging microscopy (Flowcam Fluid Imaging Technologies, Scarborough, ME, USA), with the following parameters: flowrate, 0.15 mL/min; auto-image rate, 25 frames/sec, and flowcell type, FC80FV. The size range analyzed by the microflow imaging microscopy is from 1 to 100 μm.

Formulation buffer (60 mg/mL trehalose, 5.8 mg/mL NaH2PO4, 1.2 mg/mL Na2HPO4, 0.4 mg/mL polysorbate 20, pH 6.2, 0.22 µm filtered)—similar to the formulation used with bevacizumab (Avastin, Genentech Inc., South San Francisco, CA, USA)—was kindly provided by Vaida Linkuviene (Skaggs School of Pharmacy and Pharmaceutical Sciences, University of Colorado Anschultz). A 0.05 mL volume of this protein-free buffer solution was drawn into the syringes. This volume was expelled into an Eppendorf tube containing 0.95 mL of the same buffer solution for a volume of 1 mL. The solution was mixed gently and analyzed. Purified water (0.05 mL) was loaded into the hub of each needle and, after a SO-free syringe (HSW Norm-Ject) was attached, the water was expelled into an Eppendorf tube containing 0.95 mL of water to make a 1-mL sample. This solution also was mixed gently and analyzed. All measurements were performed in triplicate for each sample. Purified water was used to assure a low background particle count before sample measurement and also between each sample. Ten samples of each needle and syringe were assessed.

The number of particles was analyzed descriptively using summary measures (mean, quartiles, minimal, maximal, standard deviation and CV, and standard/average deviation). The Feltz and Miller test was used to compare the CVs. For all statistical tests, a significance level of 5% was used. Statistical analysis were performed using the statistical software R and STATA 12 (SAS Institute, Cary, NC, USA).

No humans or animals were involved in any part of this research. The only materials analyzed in all steps were syringes and needles. All methods carried out in this research were in accordance with relevant guidelines and regulations applied to all types of research, including the ones that do not involve animals or humans subjects, like this one.

### Ethics approval and consent to participate

Obtained (CEP UNIFESP nº 2660310319).

## Data Availability

The datasets used and/or analyzed during the current study are available from the corresponding author on reasonable request.
